# Supplementary material for: Rapid resistance evolution against phage cocktails
Source: ISME J. 2026 Mar 28;20(1):wrag067. doi: 10.1093/ismejo/wrag067 (PMC13196591; doi:10.1093/ismejo/wrag067)
Supplement: SI_Rapid_resistance_evolution_against_phage_cocktails_wrag067 [file si_rapid_resistance_evolution_against_phage_cocktails_wrag067.docx]

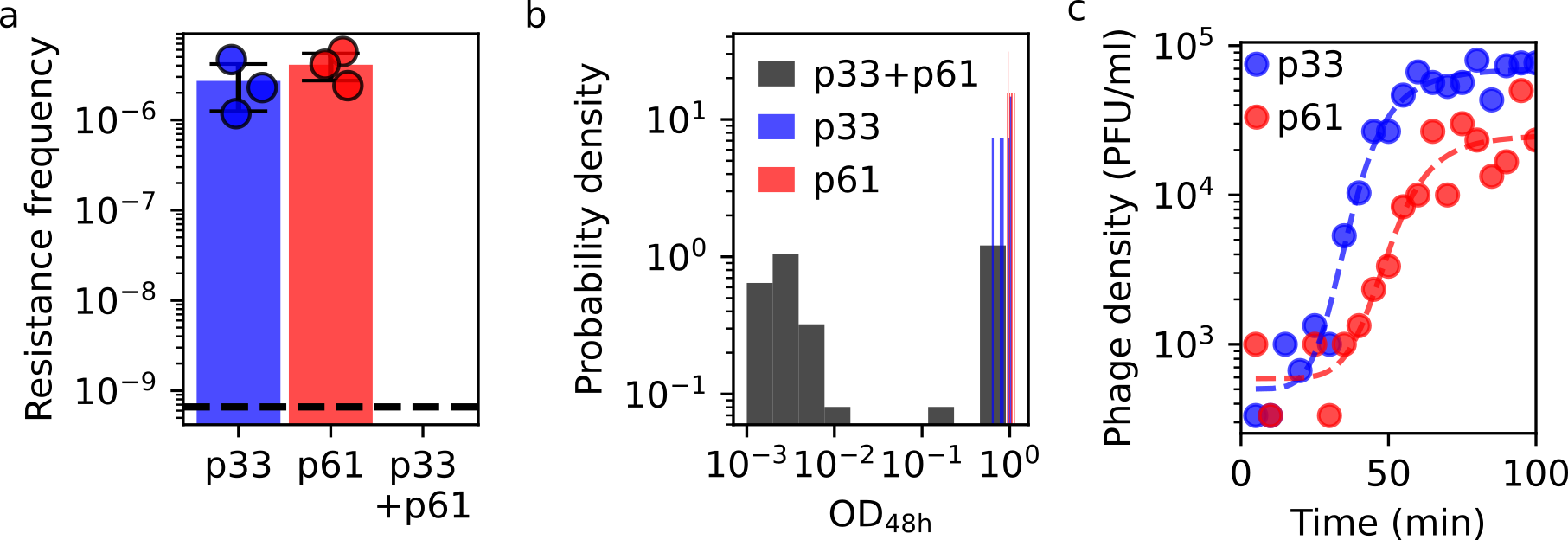


**Suppl. Fig. 1 p33 and p61 characterization. a** Resistance frequencies for p33 alone (blue), p61 alone (red), and both combined (below detection limit; dashed line). **b** Final optical density distribution after 48 h for p33 (blue), p61 (red), and the cocktail (grey). **c** One-step growth curves for p33 and p61.


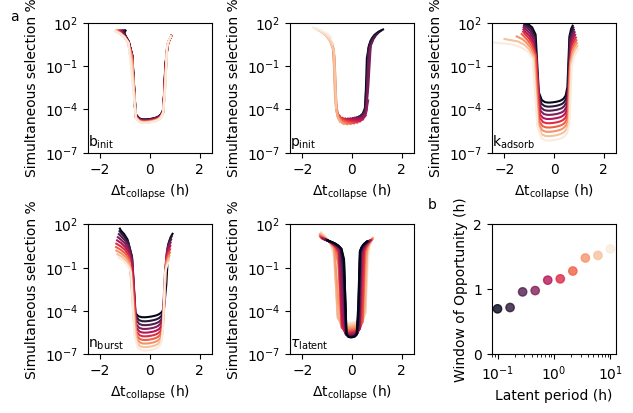


**Suppl. Fig. 2 Latent period influences the size and position of the window of opportunity to prevent resistance. a** Dual-phage simulations using identical phages, varying the inoculum concentration of one phage holding the other constant. Resistance frequency (y-axis) is plotted against the difference in collapse time (x-axis) derived from single-phage simulations. Simulations were run across a range of parameters (different panels) in logarithmic steps (ranging from low in dark to high in bright, see Suppl. Table 1 for the exact ranges). **b** Wider latent periods increase the window of collapse-time differences over which resistance is suppressed. The color coding reflects the latent period and is the same as in panel a, bottom-right.

**
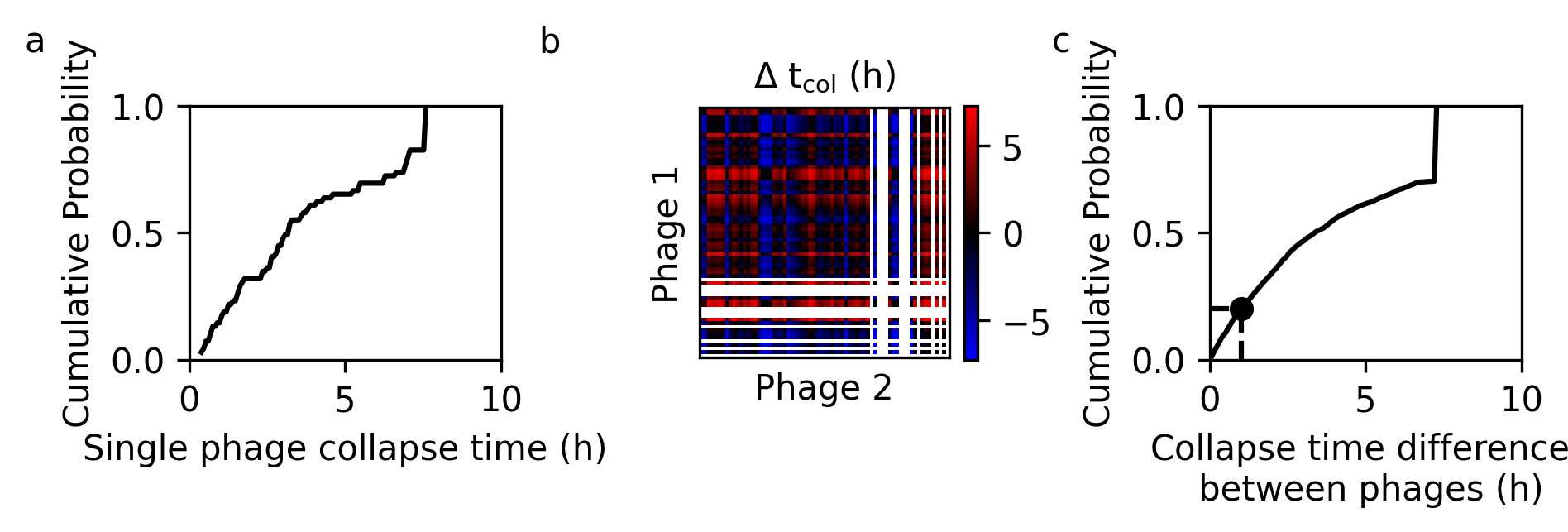
**

**Suppl. Fig. 3 BASEL collection collapse time distribution.** To estimate how often two phages might act simultaneously and thus prevent resistance, we analyzed published collapse-time data for 69 *E. coli*-infecting phages from the BASEL collection[[20]](https://www.zotero.org/google-docs/?QFFzTh). **a** Cumulative distribution of collapse times. For 11 of 69 phages, collapse times could not be measured due to very slow lysis dynamics. **b** Heatmap showing pairwise differences in collapse time for all measurable phage pairs. White lines indicate phages for which collapse times were undetermined. Collapse time differences between specific phage pairs can be found in the Supplementary Data. **c** Cumulative distribution of collapse-time differences from panel b, excluding the diagonal (self-comparisons). Pairs involving phages with indeterminable collapse times were treated as having infinite differences. ~20.0% of phage-phage pairs differ by 1 hour or less (dashed line).

**
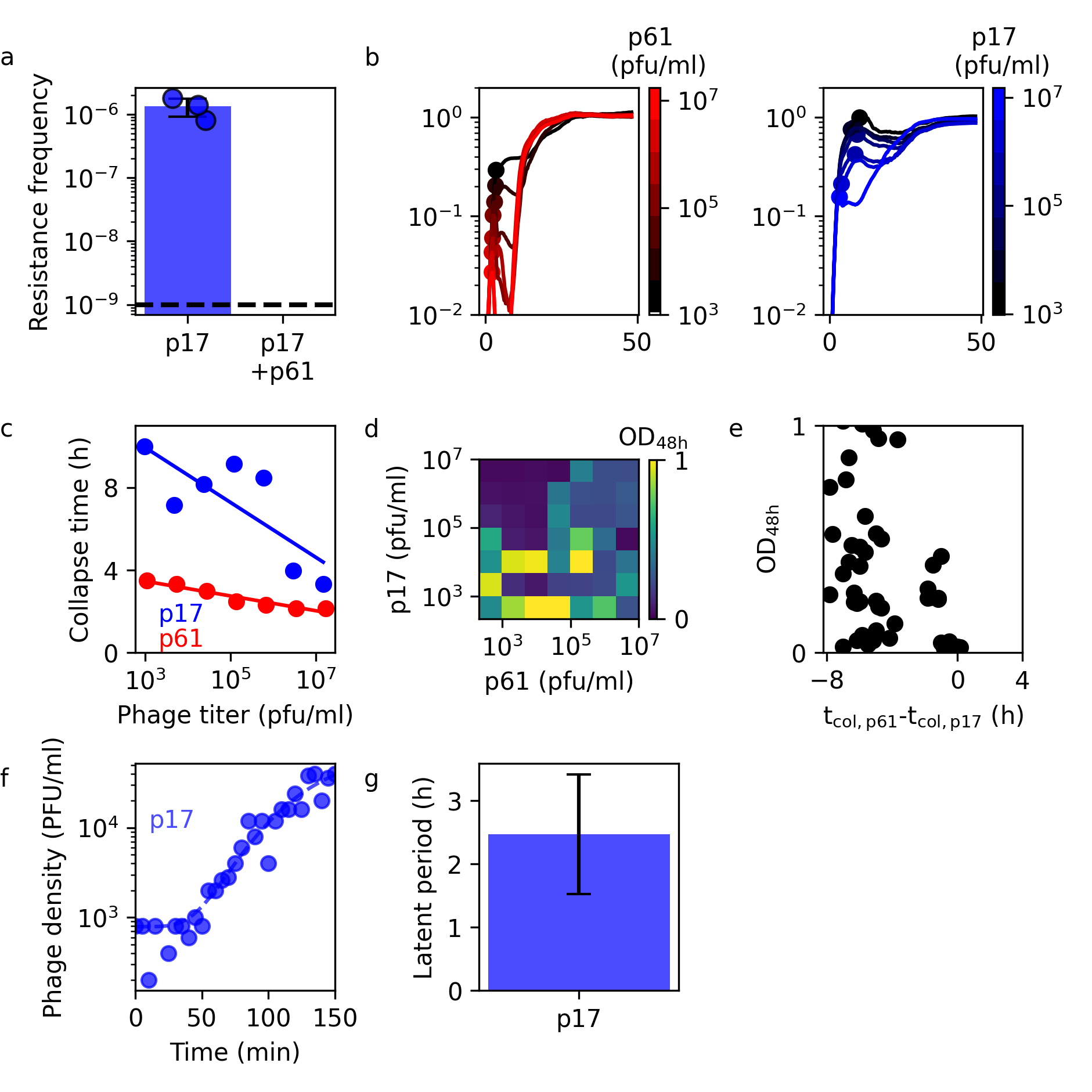
**

**Suppl. Fig. 4 phages with different bactericidal activity only show stable suppression at a large phage inoculum concentration imbalance. a** Resistance frequencies for phage 17 alone (blue) or combined with p61 (below detection limit; dashed line, see p61 alone is in Suppl. Fig 1a). The results indicate no collateral resistance between both phages. **b** Bacterial growth curves at increasing phage concentrations: left panel for p17 (blue to black), right panel for p61 (red to black). **c** Collapse times derived from growth curves in panel b. **d** Experimental data showing bacterial growth (optical density after 48 h) is only suppressed when the inoculum concentration of p17 is at least 100x higher than of p61. **e** The same data from panel c, now plotted against the difference in single-phage collapse time (panel b). **f** One-step growth curves for p17. **g** Latent period extracted from the one-step growth curve (panel f). The error bar shows the standard deviation based on the fit.

|  | [**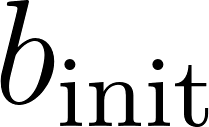**](https://www.codecogs.com/eqnedit.php?latex=b_%7B%5Cmathrm%7Binit%7D%7D#0)  (cfu) | [**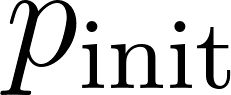**](https://www.codecogs.com/eqnedit.php?latex=p_%7B%5Cmathrm%7Binit%7D%7D#0)(pfu) | [**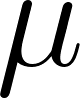**](https://www.codecogs.com/eqnedit.php?latex=%5Cmu#0)  (h^-1^) | [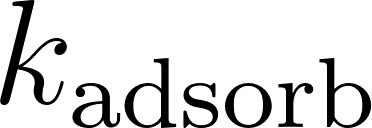](https://www.codecogs.com/eqnedit.php?latex=k_%7B%5Ctextrm%7Badsorb%7D%7D#0)  (h^-1^ cfu^-1^) | [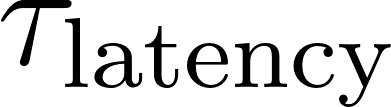](https://www.codecogs.com/eqnedit.php?latex=%5Ctau_%7B%5Cmathrm%7Blatency%7D%7D#0)  (h) | [**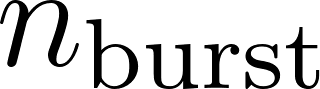**](https://www.codecogs.com/eqnedit.php?latex=n_%7B%5Cmathrm%7Bburst%7D%7D#0)  () | [**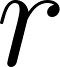**](https://www.codecogs.com/eqnedit.php?latex=r#0)  () |
| --- | --- | --- | --- | --- | --- | --- | --- |
| Fig. 1 | 10^5^ | 10^1^-10^7^ | 1.0 | 10^-8^ | 1.00 | 100 | 10^-6^ |
| Suppl. Fig. 3  (fixed) | 10^5^ | 10^1^-10^7^ | 1.0 | 10^-8^ | 1.00 | 100 | 10^-8^ |
| Fig. 3 a, Suppl. Fig. 3  (variable) | 10^4^-10^6^ | 10^3^-10^5^ | 0.2-2.0 | 10^-7^-10^-9^ | 0.02-2.00 | 50-5000 | 10^-8^ |

**Suppl. Table 1 Model parameters used in simulations.** To explore the impact of different phage traits on resistance evolution, we simulated a dual-phage predation assay in which both phages initially share identical kinetic parameters. Each parameter was varied individually across 10 logarithmically spaced steps, keeping all others constant. To modulate the difference in collapse timing, the inoculum concentration of one phage was varied across 20 logarithmically spaced steps from 10¹ to 10⁷ pfu, keepingthe concentration of the other fixed at 10⁴ pfu.
